# Prevalence and Correlates of Psychiatric Disorders in a National Survey of Iranian Children and Adolescents

**Published:** 2019-01

**Authors:** Mohammad Reza Mohammadi, Nastaran Ahmadi, Ali Khaleghi, Seyed Ali Mostafavi, Koorosh Kamali, Mehdi Rahgozar, Ameneh Ahmadi, Zahra Hooshyari, Seyyed Salman Alavi, Parviz Molavi, Nasrin Sarraf, Seyed Kaveh Hojjat, Soleiman Mohammadzadeh, Shahrokh Amiri, Soroor Arman, Ahmad Ghanizadeh, Ahmad Ahmadipour, Rahim Ostovar, Hedayat Nazari, Seyed Hamzeh Hosseini, Atieh Golbon, Firoozeh Derakhshanpour, Ali Delpisheh, Forough Riahi, Siavash Talepasand, Azizollah Mojahed, Naser Hajian Motlagh, Aazam Sadat Heydari Yazdi, Mohammad Ahmadpanah, Reza Dastjerdi, Houshang Amirian, Alireza Armani, Parvin Safavi, Maryam Kousha, Anita Alaghmand, Mahin Eslami Shahrbabaki, Arezou Kiani, Javad Mahmoudi Gharaei, Alia Shakiba, Hadi Zarafshan, Maryam Salmanian, Eric Taylor, Eric Fombonne

**Affiliations:** 1 Psychiatry and Psychology Research Center, Tehran University of Medical Sciences, Tehran, Iran.; 2 Yazd Cardiovascular Research Center, Shahid Sadoughi University of Medical Sciences, Yazd, Iran.; 3 Department of Public Health, School of Public Health, Zanjan University of Medical Sciences, Zanjan, Iran.; 4 Department of Biostatistics, University of Social Welfare and Rehabilitation Sciences, Tehran, Iran.; 5 Department of Psychiatry, Fatemi Hospital, Ardabil University of Medical Sciences, Ardabil, Iran.; 6 Department of Child and Adolescent Psychiatry, School of Medicine, Qazvin University of Medical Sciences, Qazvin, Iran.; 7 Addiction and Behavioral Sciences Research Center, North Khorasan University of Medical Sciences, Bojnurd, Iran.; 8 Department of Psychiatry, Neuroscience Research Center, Kurdistan University of Medical Sciences, Sanandaj, Iran.; 9 Research Center of Psychiatry and Behavioral Sciences, Tabriz University of Medical Sciences, Tabriz, Iran.; 10 Behavioral Sciences Research Center, Isfahan University of Medical Sciences, Isfahan, Iran.; 11 Department of Psychiatry, Hafez Hospital, Shiraz University of Medical Sciences, Shiraz, Iran.; 12 Department of Psychiatry, Booshehr University of Medical Sciences, Khalij-e-Fars Hospital, Booshehr, Iran.; 13 Social Determinants of Health Research Center, Yasuj University of Medical Sciences, Yasuj, Iran.; 14 Department of Psychiatry, School of Medicine, Lorestan University of Medical Sciences, Khorram Abad, Iran.; 15 Psychiatry and Behavioral Sciences Research Center, Addiction Institute, Mazandaran University of Medical Sciences, Sari, Iran.; 16 Department of Psychiatry, Faculty of Medicine, Hormozgan University of Medical Sciences, Bandar Abbas, Iran.; 17 Golestan Psychiatric Research Center, Golestan University of Medical Sciences, Gorgan, Iran.; 18 Department of Clinical Epidemiology, Faculty of Medicine, Psychosocial Injuries Research Center, Ilam University of Medical Sciences, Ilam, Iran.; 19 Department of Psychiatry, Golestan Educational Hospital, Faculty of Medicine, Ahvaz Jundishapur University of Medical Sciences, Ahvaz, Iran.; 20 Department of Educational Sciences and Development of Handicapped Children, Semnan University, Semnan, Iran.; 21 Health Promotion Research Center, Department of Clinical Psychology, Zahedan University of Medical Sciences, Zahedan, Iran.; 22 Alborz University of Medical Sciences, Karaj, Iran.; 23 Psychiatry and Behavioral Sciences Research Center, Mashhad University of Medical Sciences, Mashhad, Iran.; 24 Research Center for Behavioral Disorders and Substance Abuse, Hamadan University of Medical Sciences, Hamadan, Iran.; 25 Birjand University of Medical Sciences, Birjand, Iran.; 26 Department of Psychiatry, School of Medicine, Kermanshah University of Medical Sciences, Kermanshah, Iran.; 27 Department of Psychiatry, Zanjan University of Medical Sciences, Zanjan, Iran.; 28 Clinical Research Development Unit, Hajar Hospital, Shahrekord University of Medical Sciences, Shahrekord, Iran.; 29 Department of Pediatric Psychiatry, Faculty of Medicine, Guilan University of Medical Sciences, Rasht, Iran.; 30 Department of Psychiatric, Arak University of Medical Sciences, Arak, Iran.; 31 Department of Psychiatry, Neuroscience Research Center and Institute of Neuropharmachology, Kerman University of Medical Sciences, Kerman, Iran.; 32 Department of Psychiatry, Urmia University of Medical Sciences, Urmia, Iran.; 33 Institute of Psychiatry, King’s College Hospital, London, United Kingdom.; 34 Department of Psychiatry, Oregon Health and Science University, Portland, United States of America.

**Keywords:** *Comorbidity*, *Child and Adolescent*, *Epidemiology*, *Psychiatric Disorders*, *Risk Factors*

## Abstract

**Objective:** Considering the impact of rapid sociocultural, political, and economical changes on societies and families, population-based surveys of mental disorders in different communities are needed to describe the magnitude of mental health problems and their disabling effects at the individual, familial, and societal levels.

**Method** **:** A population-based cross sectional survey (IRCAP project) of 30 532 children and adolescents between 6 and 18 years was conducted in all provinces of Iran using a multistage cluster sampling method. Data were collected by 250 clinical psychologists trained to use the validated Persian version of the semi-structured diagnostic interview Kiddie-Schedule for Affective Disorders and Schizophrenia-PL (K-SADS-PL).

**Results: **In this national epidemiological survey, 6209 out of 30 532 (22.31%) were diagnosed with at least one psychiatric disorder. The anxiety disorders (14.13%) and behavioral disorders (8.3%) had the highest prevalence, while eating disorders (0.13%) and psychotic symptoms (0.26%) had the lowest. The prevalence of psychiatric disorders was significantly lower in girls (OR = 0.85; 95% CI: 0.80-0.90), in those living in the rural area (OR = 0.80; 95% CI: 0.73-0.87), in those aged 15-18 years (OR = 0.92; 95% CI: 0.86-0.99), as well as that was significantly higher in those who had a parent suffering from mental disorders (OR = 1.96; 95% CI: 1.63-2.36 for mother and OR = 1.33; 95% CI: 1.07-1.66 for father) or physical illness (OR = 1.26; 95% CI: 1.17-1.35 for mother and OR = 1.19; 95% CI: 1.10-1.28 for father).

**Conclusion: **About one fifth of Iranian children and adolescents suffer from at least one psychiatric disorder. Therefore, we should give a greater priority to promoting mental health and public health, provide more accessible services and trainings, and reduce barriers to accessing existing services.

There is a continuous need for population-based surveys on mental disorders in different communities to understand the size of the problem and of the disabilities as well as the nature of mental disorders and comorbidities and to improve the classification criteria and diagnostic instruments. Descriptive and analytic epidemiological studies can significantly help to explore psychosocial issues, genetic markers, developmental pathways, and elucidate psychopathological processes and risk mechanisms underlying psychopathology (1, 2). Given the magnitude of child and adolescent mental health needs, there is a high demand for clinical centers with expertise in managing psychiatric disorders to provide more effective and reasonable services. Clearly, data are required to guide policy makers and assist planning mental health services for the youth (3-6). The prevalence of psychiatric disorders in children and adolescents has attracted much attention around the world (7) and the number of such studies has grown substantially over the last three decades (8-19). For instance, Goodman et al. (20) have reported a rate of 15.3% of any ICD-10 psychiatric disorders among 7-14 year-old participants in their school-based survey conducted in a populous city of Russia. Anselmi et al. (21) have conducted a population-based survey among 11-12 year-old Brazilian individuals. This non-national wide survey has been administered using the Strengths and Difficulties Questionnaire (SDQ), and the authors reported a prevalence of psychiatric disorders to be 10.8% in the preadolescent population. Benjet et al. (22) conducted a household-based survey among the adolescents residing in Mexico City.

They reported a 12-month prevalence of 40% in their samples. Furthermore, few but valuable national surveys with larger population have been performed in some countries.

Kessler et al. (23) have designed a household- and school-based survey among the 13-17 year-old American adolescents. They reported a 12-month prevalence of any DSM-IV disorder of 40.3% and a one-month prevalence of 23.4% in their samples. In a population-based survey of British children and adolescents, Ford et al. (24) reported the overall prevalence of DSM-IV disorders of 9.5% using the Development and Well-Being Assessment (DAWBA). Vicente et al. (25) have reported a 12-month prevalence of 22.5% among 4-18 year-old Chileans in their national household-based survey.

Despite valuable studies conducted in different parts of the world, we may not find many psychiatric epidemiological studies (26-29) in the Middle East as an important transcontinental region, particularly in Iran. Eapen et al. (30) have conducted a small survey on 620 individuals aged 6 to 18 years in a populous city of the United Arab Emirates (UAE) and reported a 22.2% prevalence rate of DSM-IV disorders.

In another study in this region, Alyahri and Goodman (31) have designed a non-national wide survey to estimate the prevalence of mental disorders among 7-10 year-old Yemeni schoolchildren and reported the overall prevalence of 15.7% in their samples (n = 1210). Also, our research group in the previous study (32) has conducted a psychiatric epidemiological survey of Iranian children and adolescents in 5 populous cities. The results have shown that the overall prevalence of psychiatric disorders was 10.55%. These studies have not been performed at national or international level and they often comprised small samples of adolescent population. Therefore, they cannot be a representative study for the status of psychiatric disorders among children and adolescents in the region. 

Thus, we can conclude that there is a need to conduct a large population-based survey among children and adolescents to draw the profile of children and adolescents’ mental disorders in the Middle East, particularly in Iran as an important country in this region. The purpose of the present study was as follows: (1) to find the prevalence of specific psychiatric disorders in Iranian children and adolescents (IRCAP Study) and use epidemiologic approaches to gain more insight into adolescents’ psychiatric disorders in the Middle East (2) to build a national database on all of the psychiatric disorders in Iranian children and adolescents using valid and reliable assessment tools, (3) to identify children and adolescents at high risk of psychiatric disorders for primary prevention, and (4) to investigate the risk factors and correlates of mental disorders in a large young population**.**

## Materials and Methods

***Site***

Iran is a large country with 1.648 million km² land and near 80 million inhabitants. Iran consists of 31 provinces. About 74% of Iran’s population is living in urban areas and 26% in rural areas, 50.7% is male and 49.3% is female. The child and adolescent population (5-19 year-old) of Iran is near 17.5 million (21.9%). Literacy rate among the population aged 6 and above is 87.6% (33). Moreover, 58% of disability-adjusted life years (DALYs) at the national level in Iran is due to non-communicable diseases, meanwhile depression is the seventh cause of DALYs (34).

***Study Design***

The Iranian Children and Adolescents' Psychiatric Disorders Study (IRCAP) was the first national community-based cross sectional study implemented in all provinces of Iran by the Psychiatry and Psychology Research Centre located in Roozbeh hospital, Tehran, Iran. This study was a population-based cross sectional survey of 30 532 children and adolescents aged 6- 18 years from all provinces of Iran. The trained interviewers referred to the houses, which were randomly selected considering the postal code using a multistage cluster sampling method. The study objectives and procedures were explained to those interviewed and the parents and children were invited to participate in the survey. The study protocol and methodology have been previously reported in detail by Mohammadi et al. (35). 

***Sampling***

The samples in the IRCAP study, as inferred above, were randomly selected using the multi-stage stratified cluster random sampling method from among the 6-18-year-old population living in urban and rural areas (proportional to population rate) of all 31 provinces of Iran. In each province, 170 clusters of houses were randomly selected based on postal codes in both rural and urban areas; and in each cluster, 6 children and adolescents were randomly selected within equal blocks of gender and age groups (6-9 years, 10-14 years, and 15-18 years). Then, about 1000 children and adolescents were selected in each province except for Tehran, which included 340 clusters and 2040 children and adolescents. The administrating team of investigation tried to address the sources of bias in the design of the study, from the very beginning to the end. At the design stage, it was tried to decrease the selection bias with the use of the multistage cluster sampling and stratified random sampling methods. Also, it was tried to distribute the main confounders, such as age and sex, evenly in each cluster.

***Study Locations***

 Thirty-one study sites were established across the country in each provincial capital under the supervision of the psychiatry and psychology research center, Roozbeh hospital, Tehran, Iran. The study fields included 31 urban areas and 636 rural areas.

***Participants***

All Iranian citizens aged 6-18 years were eligible to participate in this national survey. To minimize the effects of migration on the sociocultural characteristics, the exclusion criterion included non-Iranian citizens and immigrants. Furthermore, those who did not consent to participate were excluded. 

***Instruments***

To identify psychiatric disorders in the screening and diagnostic stage, the participants were surveyed using the K-SADS-PL measures whose validity and reliability has been first verified by Joan Kaufman et al. The test-retest reliability was in the range of 0.77 to 1.00 for present and lifetime diagnosis of different psychiatric disorders, and the level of inter-rater agreement in diagnosing the disorders was between 93% to 100% (36). Ahmad Ghanizadeh et al. assessed the reliability and validity of the Persian version of K-SADS-Pl in Iran (37). They reported the test-retest reliability of 0.56-0.81 to diagnose different psychiatric disorders, and the inter-rater reliability was at the level of 0.69. Also, the basic sociodemographic information was gathered by a questionnaire devised particularly for this study. The demographic and familial characteristics included sex, age, residential area, parental education levels, parental job, and a history of parental physical or mental illness, the data about which were gathered via self-report using a semi-structured questionnaire. The parents’ health components show whether the parents had a history of any psychiatric or physical illnesses leading to referral to a clinic or specialist, treatment, or hospitalization. Both parents and children asked to participate into the interviews simultaneously. Moreover, other reliable tools were used to discriminate epilepsy, mental retardation, and tobacco use.

***Interviewer Training and Data Collection***

Data collection was done by 250 trained clinical psychologists across the country referring to houses from September 22, 2016 through January 3, 2018. Before the survey, all interviewers across the country were invited to participate in a training course of K-SADS-PL, taught by a psychiatrist. The interviewers were also trained to explain the study process to the interviewees and ensure the confidentiality of the information gathered to guarantee the maximum collaboration of the households. In this study, the kids as well as their parents, especially the mothers, were interviewed, and a summary rating was made by the both interviewers to make a best-estimate diagnosis using DSM-IV. Furthermore, in the study period, the administrative team implemented an auditing process to inspect the interviewers by travelling to all provinces across the country for closer inspections. Also, the auditors were randomly calling the members of the sample population during the study to double-check the precision of interviews and data gathering process. Furthermore, in the data gathering and implementation stage, it was tried to reduce the information bias through employing the trained psychologists across the country to complete the validated Persian version of K-SADS-PL based on the DSM-IV diagnostic criteria. The sensitivity and specificity of the Persian version of K-SADS are shown to be high (37). In our study, each interview lasted for 30 to 90 minutes, depending on the interviewee’s conditions. The inter-rater coefficient between the interviewers was found to be 0.91 (p < 0.001).

***Statistical Analysis***

The collected data were analyzed from April 9, 2018 through May 25, 2018. First, the data collected from all provinces of the country were screened and cleaned to find any wrong or unexpected data that may have occurred in data entry. Frequency statistics and percentages were utilized to obtain the prevalence data in terms of demographic variables. All the statistics on the prevalence of psychiatric disorders among children and adolescents were also based on the weighted percentages (weighting based on the number of provincial samples) as well as crude percentages (without weight). The post-stratification weights to adjust the survey sample to the underlying population demographic structure. The weighted percentages were measured based on the population distribution of children and adolescents across each province according to the 2017 national census (1 weight was considered as criterion for every 1 million people and the other weights were determined accordingly). Furthermore, the Binary Logistic Regression was used to predict the probability of any specific psychiatric disorder by considering the independent variables as predictors. In the first step, we used the univariate model to determine the significant predictive variables, and in the second step those independent variables with significant ORs added to the multivariate model (95% confidence interval (CI) was used to estimate the precision of the ORs).

In addition to using the power of random sampling in controlling unknown confounding factors, we tried to decrease the confounding bias in the analysis stage by categorizing the known confounders and by using the multivariate analysis via adjusting the effects of confounding variables.

***Ethics***

Written consent was obtained from the parents of younger than 15 participants and from the participants and their parents of adolescents aged 15 to 18 years. All information about the participants and their families were kept confidential. Moreover, those children and adolescents who were diagnosed with a disorder in this study were treated and managed free of charge by one of the child and adolescent psychiatrists who participated in the project. The Ethics Committee Board of the National Institute for Medical Research Development (NIMAD) has approved this study (the ethics code: IR.NIMAD.REC.1395.001).

## Results

From a total of 33 264 children and adolescents aged 6-18 years who were invited to participate in this study, 30 532 consented to participate in this survey (response rate: 91.78%). Of them, 15 618 (51.2%) were females and 14 914 were (48.8%) males. The average age of the participants was 11.81±3.78 (11.76 ± 3.81 in females and 11.87±3.75 in males); 83.5% of them lived in urban areas and others (16.5%) lived in rural areas.

The total prevalence of psychiatric disorders in children and adolescents was 22.31% (95% CI: 21.81-22.82), meaning that 6209 children and adolescents were diagnosed with at least one psychiatric disorder. However, the reported percentage was based on the weighted percentages and the report on the total prevalence of psychiatric disorders did not include mental retardation, epilepsy, and tobacco use. The variables in Table 1 are included in two general classes: the demographics and the parents’ health status. The table shows a higher prevalence of psychiatric disorders among those children and adolescents whose parents had a positive history of mental and physical illnesses.

**Figure 1:**
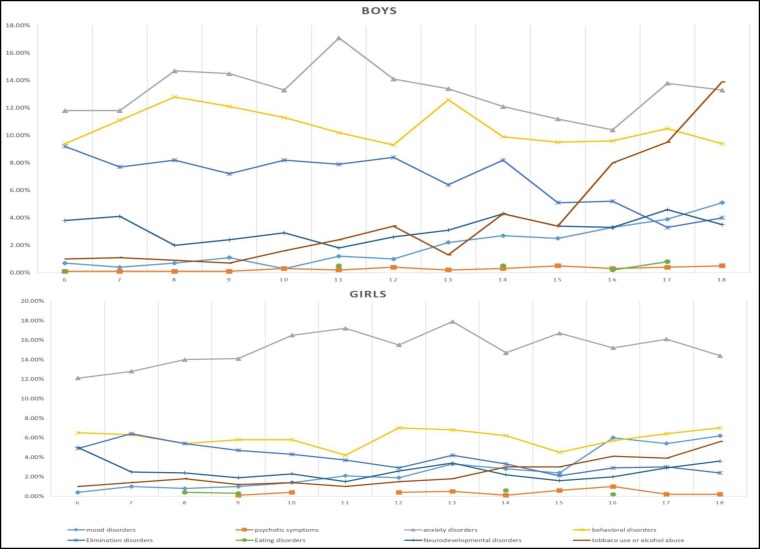
Sex Difference in the Rate of Iranian Children and Adolescents Psychiatric Disorders in each Year of Life

As shown in Table 1, the total prevalence of psychiatric disorders among males (weighted percentage) was 24.04% (23.36-24.73), and it was 20.62% (19.99-21.26) among females (OR = 0.85; 95% CI: 0.80-0.89). The difference in the prevalence of psychiatric disorders between the two genders was statistically significant (p = 0.001). Figure 1 shows the sex difference in the rate of Iranian Children and Adolescents Psychiatric Disorders in each year of life. The prevalence of psychiatric disorders in children and adolescents living in urban areas was 23.0% (22.49-23.52), while it was 16.8% (15.79-17.86) in children and adolescents living in rural areas (OR = 0.80; 95% CI: 0.73-0.87).

Also, the higher the level of parents’ education (whether father or mother), the lower the probability of psychiatric disorders in children and adolescents. Additionally, parental history of physical and mental illnesses increases the probability of having children with psychiatric disorders. Compare to physical illness, the increase in the odds of a psychiatric disorder in the child was larger for both mother (OR = 1.96 vs OR = 1.26) and father (1.33 vs 1.19) (Table 1).

**Figure 2:**
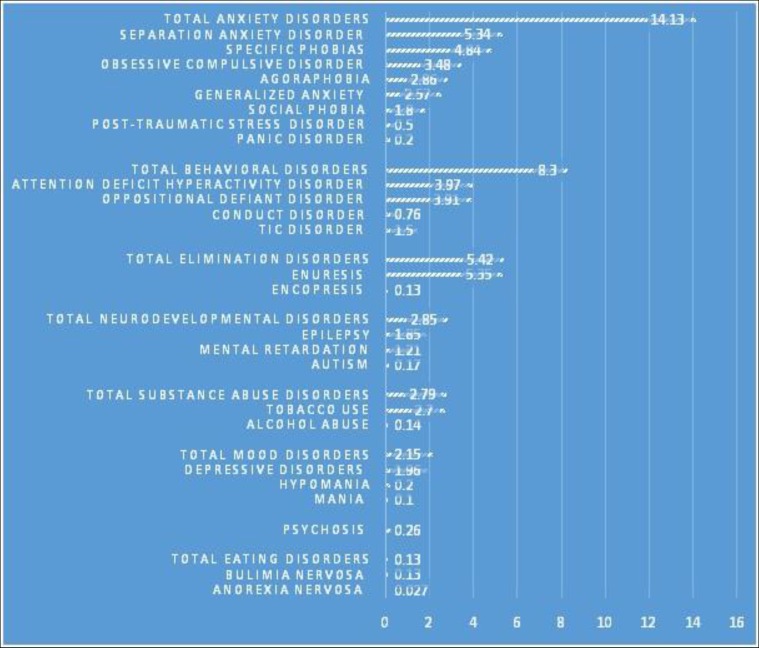
Percent of Psychiatric Disorders in Children and Adolescents

Table 2 displays the prevalence of psychiatric disorders and their 95% confidence intervals in children and adolescents. Anxiety disorders had the highest prevalence (14.13%), and the separation anxiety disorder (5.34%) was the most common subtype in this class. The frequencies and percentages (95% CI) of other psychiatric disorders are presented in Table 2 and Figure 2. Figure 1 shows the differences in the frequency of psychiatric disorders in children and adolescents in each age group according to sex.

**Figure 3:**
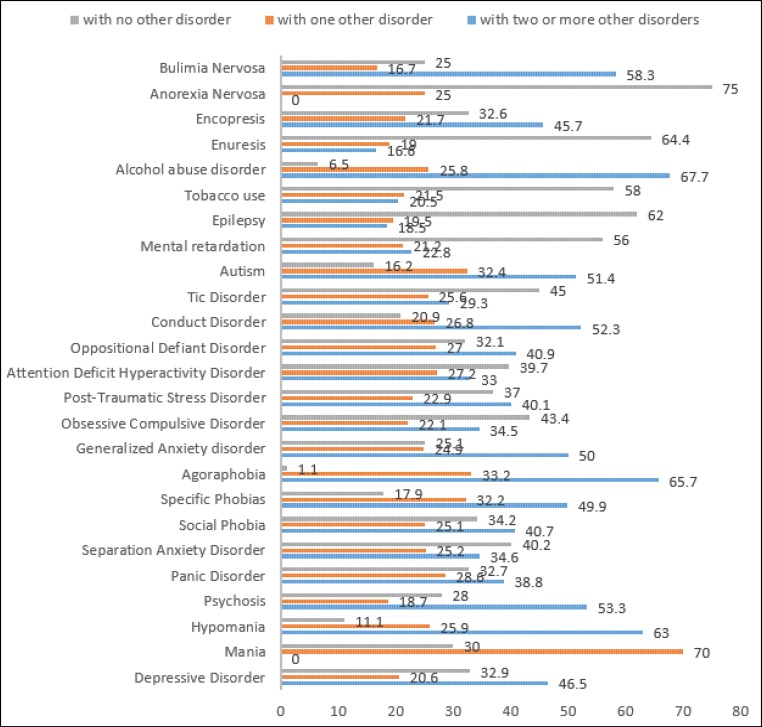
The Co-Morbidity Graph of Psychiatric Disorders in Children and Adolescents with One or More Other Psychiatric Disorder

Table 3 shows the comorbidity of psychiatric disorders with each other in children and adolescents. Results revealed that the comorbidity of mood disorders with anxiety disorders (51.1%) and behavioral disorders (37.9%) was more than other comorbidities. The graph of the comorbidity of psychiatric disorders in children and adolescents with one or more psychiatric disorders is illustrated in Figure 3.

## Discussion

The purpose of this study was to determine the prevalence and comorbidity of psychiatric disorders among 6-18-year-old children and adolescents across the country. The high response rate of this survey indicated that children’s behavior and emotions are very important issue for the community and parents. The total prevalence of psychiatric disorders among children and adolescents was 22.31%. This result is comparable to the findings of similar studies that have been conducted in Germany and Switzerland (38). However, some differences were observed that may be due to socioeconomic issues, the instruments used, the sample size, and sampling method, the subjectivity of interviewers, and the location and time of the survey. In the previous survey of psychiatric disorders among children and adolescents in Iran, which has been conducted in a small scale, the general rate of occurrence of psychiatric disorders was reported to be 10.55% (32). Therefore, an increasing trend was observed in the prevalence of mental disorders among Iranian children and adolescents. Psychiatric disorders grouped in the category of anxiety disorders with a prevalence of 14.13% were the most frequent mental disorders among all children and adolescents; this category included the separation anxiety (5.34%), special phobia (4.84%), obsessive compulsive disorder (3.48%), agoraphobia (2.86%), general anxiety (2.57%), social phobia (1.8%), posttraumatic stress disorder (0.5%), and panic disorder (0.2%). This finding is consistent with the results of a recent systematic review on prevalence of anxiety disorders among children and adolescents in Iran (39). The second most prevalent disorder in children and adolescents was behavioral disorders, particularly attention deficit hyperactivity disorder (ADHD) and oppositional defiant disorder (ODD).

Our results indicated that the factors of gender and place of residence play an important role in the epidemiology of psychiatric disorders in children and adolescents. In this work, we observed that disorders, such as ADHD, enuresis and autism are more prevalent in males than in females, particularly in childhood. Therefore, childhood psychiatric disorders were more prevalent in males than in females. In addition, psychiatric disorders were more prevalent among 6-12-year-old males compared to females; but their prevalence among 12-18-year olds was approximately the same between males and females (although at the age of 14, males showed a higher prevalence of the disorders again). Furthermore, psychiatric disorders were significantly more prevalent among children and adolescents in urban areas than in rural areas. The stresses, concerns, and the stimuli that affect individuals in large cities and the challenges that these individuals had to cope with resulted in a higher prevalence of psychiatric disorders in urban settings than in rural environments (40). On the other hand, the issues that can affect children’s mood are more diverse in urban areas than in rural areas, and due to this diversity of such events, individuals living in cities more often experience failure than those who live in rural places. This experience of failure has an effect on the incidence and exacerbation of psychiatric disorders. Previous studies have also reported a higher prevalence for psychiatric disorders and the risk of suicide in urban areas among Danish and Mexican-American populations (40, 41).

As previous studies have reported (42), low maternal education is one of the most significant risk factors associated with the child and adolescent psychiatric disorders. On the other hand, it was observed that as the level of the father’s education increased, the incidence of psychiatric disorders in children decreased. Moreover, as expected, a history of any physical and mental illness in parents can increase the incidence of psychiatric disorders in children and adolescents. In fact, the parental health variables can be good predictors of children and adolescents’ psychiatric disorders. This finding is consistent with previous studies that have reported the relationship between psychiatric disorders in children and somatization symptoms in their mothers (43). Meanwhile, the mental illness of the mother had the largest odds ratio, followed by the mental illness of the father, maternal physical illness, and the physical illness of the father.

Analyses of comorbidities in this research have yielded interesting results. At first, anxiety disorders had the highest comorbidity with other disorders. Previous studies confirm this finding by assessing comorbidities in mood disorder, psychotic disorder, alcohol use, eating disorder, and anxiety disorders (44-50). Results revealed that children and adolescents with mood disorders were highly vulnerable to anxiety and behavioral disorders as the most probable comorbidities. The results further showed that in 32.9% of cases depression did not occur in comorbidity with other disorders, but it was the only disorder in the child or adolescent. However, 20.6% of children with depression had 1 comorbid disorder and 46.5% of them had 2 or more comorbidities. The results of the study conducted by Noterdaeme et al. were also consistent with these results. Their results showed that more than 60% of the patients were diagnosed with more than one psychiatric disorder (51). Nearly half of the children with psychosis had 2 or more comorbid disorders. Among the anxiety disorders, agoraphobia, generalized anxiety, and specific phobia were, in most cases, comorbid with 2 or more other disorders. Among behavioral disorders, the conduct disorder had a higher comorbidity with other disorders, while the tic disorder was often observed as a single disorder. Among neurodevelopmental disorders, autism was, in most cases, comorbid with other disorders. Also, participants with alcohol abuse often had other comorbid disorders. Furthermore, the age and gender variables showed that individuals with mood disorders comorbid with behavioral disorders might be females with only a very small probability and that the elimination disorder cannot be expected in adolescents (15-18 years) with mood disorders. Moreover, adolescents with mood disorders were considerably vulnerable to smoking and alcohol abuse disorder. Children and adolescents with psychotic disorders were highly vulnerable to behavioral and anxiety disorders as the most probable comorbidities. Children and adolescents with anxiety disorders were highly vulnerable to behavioral and elimination disorders as the most probable comorbidities. Those 10-18 years of age with anxiety disorders were meaningfully vulnerable to comorbid mood disorders, with the probability being much higher in adolescents. This was also true about the elimination disorder. For more details about the comorbidity of psychiatric disorders among children and adolescents based on age and gender see Table 3.

## Limitation

The IRCAP study was the first epidemiological survey of psychiatric disorders with a large sample of children and adolescents living in Iran. However, several limitations apply when interpreting its findings. First, the IRCAP was conducted in state capitals only, and the other cities and towns in the provinces were not surveyed. Second, the IRCAP did not survey the detained or homeless children and adolescents. Third, the survey gathered data about the social and familial factors in a self-report manner. As a result, responses might have been affected by inaccurate recall of the events. Moreover, in these situations, people usually answer the questions in the interview cautiously because they don't trust the interviewers. However, our interviewers were trained to minimize this limitation.

## Conclusion

About one fifth of Iranian children and adolescents suffer from at least 1 psychiatric disorder. Psychiatric disorders of the children and adolescents significantly differ in terms of sex, place of residence, parental education, and health variables. Anxiety disorders and behavioral disorders are the most prevalent disorders and should be considered in future etiological and interventional studies. Importantly, adolescents with any psychiatric disorder are considerably vulnerable to smoking and substance abuse disorders. Indeed, the high prevalence of these disorders justifies the special attention of public and clinical health systems to the mental health status of children and adolescents, especially those with low social and familial indicators living in urban areas. Policymakers in the field of mental health should set new goals based on the data provided and plan targeted programs. In particular, careful premarital screening should be done for physical and mental illnesses, and appropriate health education programs should also be provided. Moreover, we need to increase awareness and insight in those parents who have a low education level and have a history of mental disorders. 

Considering the large sample size collected in this study, a nearly comprehensive database of psychiatric profiles of Iranian children and adolescents was obtained that can be used for many clinical and research purposes in the future.

**Table 1:** Prevalence of Psychiatric Disorders in the Iranian Children and Adolescents and Association with Demographic and Family Variables (N =30,532)

**Socio-Demographic Characteristics**			**Total**	**With Disorders**		**Univariate ** **Analysis**	**Multivariate ** **Analysis**
			**N (%)**	**N (Unweighted %)**	**Weighted % (95% CI)**		
						**OR (CI 95%)**	**OR (95% CI)**
Gender	Boy		14914 (48.8)	3253 (21.8)	24.04 (23.36-24.73)	Referent	
	Girl		15618 (51.2)	2956 (18.9)	20.62 (19.99-21.26)	0.84 (0.79-30.88)**	0.85(0.80-0.90)**
Age	6-9		10466 (34.3)	2158 (20.6)	22.02 (21.24-22.82)	Referent	
	10-14		10655 (34.9)	2182 (20.5)	23.10 (22.31-23.91)	0.99 (0.93-1.06)	0.96(0.89-1.03)
	15-18		9409 (30.8)	1869 (19.9)	21.70 (20.88-22.54)	0.95 (0.89-1.02)	0.92(0.86-0.99)*
Residential area	Urban		25493 (83.5)	5292 (20.8)	23.00 (22.49-23.52)	Referent	
	Rural		5037 (16.5)	917 (18.2)	16.80 (15.79-17.86)	0.85 (0.78-0.92)**	0.80(0.73-0.87)**
Father educations	Illiterate		1311 (4.5)	277 (21.1)	21.36 (19.23-23.66)	Referent	
	primary school		4723 (16.1)	1002 (21.2)	23.73 (22.54-24.96)	1.00 (0.86-1.17)	0.93 (0.79-1.09)
	Guidance & high school		6546 (22.3)	1446 (22.1)	24.12 (23.10-25.17)	1.06 (0.92-1.22)	0.90 (0.76-1.06)
	Diploma		8537 (29.1)	1700 (19.9)	22.21 (21.34-23.10)	0.93 (0.81-1.07)	0.75 (0.63-0.89)**
	bachelor		6184 (21.1)	1162 (18.8)	21.10 (20.09-22.12)	0.86 (0.75-1.00)	0.69 (0.58-0.83)**
	MSc or higher		2008 (6.9)	392 (19.5)	21.77 (20.01-23.62)	0.91 (0.76-1.08)	0.75 (0.61-0.93)**
	Missing		1225	230			
Mother educations	Illiterate		1727 (5.8)	330 (19.1)	20.26 (18.44-2.23)	Referent	
	primary school		5577 (18.8)	1150 (20.6)	22.00 (20.93-23.11)	1.10 (0.96-1.26)	1.14 (0.98-1.33)*
	Guidance & high school		5811 (19.6)	1242 (21.4)	23.78 (22.70-24.89)	1.15 (1.00-1.32)*	1.23 (1.05-1.44)*
	Diploma		9825 (33.2)	2059 (21.0)	23.24 (22.42-24.09)	1.12 (0.99-1.28)*	1.27 (1.08-1.49)**
	Bachelor		5669 (19.1)	1115 (19.7)	21.94 (20.88-23.04)	1.04 (0.90-1.19)	1.23 (1.03-1.46)*
	MSc or higher		1006 (3.4)	174 (17.3)	21.00 (18.57-23.59)	0.88 (0.72-1.08)	1.02 (0.80-1.30)
	Missing		919	139			
Father Occupation(1)	unemployed		1007 (3.4)	219 (21.7)	21.00 (18.55-23.57)	$X^{2}$X^2^ = 20.30 P = 0.005	
	Labourer		16812 (57.2)	3461 (20.6)	22.33 (21.71-22.97)		
	Farmer		994 (3.4)	194 (19.5)	18.65 (16.31-21.10)		
	businessman		1085 (3.6)	240 (22.1)	25.63 (23.11-28.30)		
	Retired		1716 (5.8)	382 (22.3)	23.74 (21.77-25.79)		
	public sector		6759 (23.0)	1322 (19.6)	23.19 (22.19-24.20)		
	Teacher		820 (2.8)	141 (17.2)	20.00 (17.40-22.87)		
	faculty member		176 (0.6)	33 (18.8)	12.43 (8.40-18.20)		
	Missing		1165	217			
Mother Occupation(2)	Laborer		1016 (3.5)	218 (21.4)	24.81 (22.20-27.50)	X^2 ^ $X^{2}$= 7.50 P = 0.276	
	Tradesman		229 (0.7)	42 (18.3)	17.80 (13.48-23.38)		
	Housewife		25336 (85.2)	5225 (20.6)	22.30 (21.79-22.82)		
	Retired		222 (0.7)	49 (22.1)	27.36 (22.03-33.70)		
	Employee		1661 (5.6)	318 (19.1)	21.55 (19.64-23.59)		
	Teacher		1185 (4.0)	228 (19.2)	21.95 (19.68-24.38)		
	Faculty Member		75 (0.3)	12 (16.0)	15.52 (9.40-25.92)		
	Missing		810	117			
History of parents physical illness	Father	No	25532 (84.3)	5009 (19.6)	21.72 (21.22-22.23)	Referent	
		Yes	4745 (15.7)	1136 (23.9)	25.40 (24.18-26.66)	1.29 (1.20-1.39)**	1.19 (1.10-1.28)**
		missing	257	64			
	Mother	No	24695 (82.2)	4761 (19.3)	21.03 (20.53-21.54)	Referent	
		Yes	5349 (17.8)	1320 (24.7)	27.40 (26.23-28.62)	1.37 (1.28-1.47)**	1.26(1.17-1.35)**
		missing	490	128			
History of parents mental illness	Father	No	30049 (98.6)	6063 (20.2)	22.18 (21.71-22.65)	Referent	
		Yes	434 (1.4)	128 (29.5)	32.50 (28.25-37.03)	1.66 (1.34-2.04)**	1.33 (1.07-1.66)*
		missing	51	18			
	Mother	No	29929 (98.2)	5989 (20.0)	22.02 (21.55-22.49)	Referent	
		Yes	541 (1.8)	197 (36.4)	36.50 (32.46-40.55)	2.29 (1.92-2.73)**	1.96 (1.63-2.36)**
		missing	64	23			
Total			30532 (100)	6209 (20.3)	22.31 (21.81-22.82)		

The total statistics does not include mental retardation, epilepsy and tobacco use. OR = Odds Ratio adjusted; CI = Confidence Interval;

* p ≤ 0.05;

** p < 0.01.

**Table2:** Prevalence of Specific Psychiatric Disorders in Iranian Children and Adolescents (N= 30,532)

**Psychiatric Disorders**		**Number**	**Crude percent**	**Weighted percent** **(CI 95%)**
Mood Disorders	Depressive Disorders	535	1.9	1.96 (1.81 - 2.13)
	Mania	30	0.1	0.1 (0.07-0.14)
	Hypomania	54	0.2	0.2 (0.16-0.26)
	**Total Mood Disorders**	566	2	2.15 (1.99-2.32)
Psychotic Disorders	Psychosis	75	0.3	0.26 (0.2-0.3)
Anxiety Disorders	Panic Disorder	49	0.2	0.2 (0.16-0.26)
	Separation Anxiety	1391	4.7	5.34 (5.09-5.6)
	Social Phobia	585	2.00	1.8 (1.66-1.96)
	Specific Phobias	923	3.1	4.84 (4.6-5.09)
	Agoraphobia	644	2.2	2.86 (2.68-3.06)
	Generalized Anxiety	746	2.5	2.57 (2.4-2.76)
	Obsessive Compulsive Disorder	747	2.5	3.48 (3.28-3.69)
	Post-Traumatic Stress Disorder	192	0.7	0.5 (0.43-0.59)
	**Total Anxiety Disorders**	3420	11.9	14.13 (13.73-14.54)
Behavioral Disorders	Attention Deficit Hyperactivity Disorder	1175	4	3.97 (3.75-4.2)
	Oppositional Defiant Disorder	1124	3.8	3.91 (3.7-4.14)
	Conduct Disorder	235	0.8	0.76 (0.67-0.87)
	Tic Disorder	351	1.2	1.5 (1.37-1.64)
	**Total Behavioral Disorders**	2320	7.9	8.3 (7.99-8.62)
Neuro-developmental disorders	Autism	37	0.1	0.17 (0.13-0.22)
	Mental retardation	377	1.3	1.21 (1.09-1.34)
	Epilepsy	569	1.9	1.85 (1.7-2.01)
	**Total Neurodevelopmental disorders**	885	3	2.85 (2.67-3.05)
Substance abuse disorders	Tobacco use	631	2.1	2.7 (2.52-2.89)
	Alcohol abuse	31	0.1	0.14 (0.1-0.19)
	**Total Substance abuse disorders**	642	2.3	2.79 (2.6-2.9)
Elimination Disorders	Enuresis	1515	5.1	5.35 (5.1-5.61)
	Encopresis	46	0.2	0.13 (0.1-0.18)
	**Total Elimination Disorders**	1535	5.2	5.42 (5.15-5.66)
Eating Disorders	Anorexia Nervosa	4	0.013	0.027 (0.025-0.029)
	Bulimia Nervosa	12	0.04	0.13 (0.1-0.19)
	**Total Eating Disorders**	16	0.052	0.13 (0.1-0.19)
Total psychiatric disorders*		6209	20.3	22.31 (21.81-22.82)

* The total statistics does not include mental retardation, epilepsy and tobacco use

**Table 3:** Comorbidity between Psychiatric Disorders in Iranian Children and Adolescents

**Comorbid** **Main**		**Mood ** **Disorders**	**Psychotic ** **Disorders** **n (%)**	**Anxiety ** **Disorders** **n(%)**	**Behavioral ** **Disorders** **n(%)**	**Neurodevelopme** **ntal disorders ** **n(%)**	**Substance ** **abuse ** **disorders ** **n(%)**	**Elimination ** **Disorders** **n(%)**	**Eating ** **Disorders** **n(%)**
Mood Disorders	n (%)		20(3.5)	289(51.1)	211(37.9)	47(8.3)	63(11.9)	50(8.9)	4(0.7)
	OR (CI 95%)		42.85(23.92- 76.78)	8.80(7.10- 10.91)	7.84(6.32- 9.72)	2.40 (1.56-3.69)	5.87(4.27- 8.06)	1.31(0.88- 1.95)	11.32(4.06 -31.54)
Psychotic Disorders	n (%)	20(26.7)		37(49.3)	39(52.7)	11(14.7)	3(4.4)	8(10.7)	0
	OR (CI 95%)			8.48(4.76- 15.10)	14.51(8.17- 25.78)	3.22(1.14-9.09)	0.68(0.08-5.5)	1.64(0.59- 4.53)	
Anxiety Disorders	n (%)	289(8.5)	37(1.1)		824(24.5)	171(5.1)	124(3.9)	332(9.8)	7(0.2)
	OR (CI 95%)				4.95(4.41- 5.55)	1.81(1.46-2.26)	1.83(1.47- 2.27)	2.07(1.78- 2.42)	10.50(4.51 -24-45)
Behavioral Disorders	n (%)	211(9.1)	39(1.7)	824(35.5)		163(7.1)	150(6.9)	298(13)	3(0.1)
	OR (CI 95%)					2.91(2.32-3.66)	3.44(2.77- 4.27)	3.12(2.65- 3.67)	1.14(0.27- 4.77)
Neurodevelop mental disorders	n (%)	47(5.3)	11(1.2)	171(21.2)	163(19.5)		37(4.6)	93(11)	0
	OR (CI 95%)						1.68(1.10- 2.57)	2.37(1.79- 3.13)	
Substance abuse disorders	n (%)	63(10.3)	3(0.5)	124(21.4)	150(24.3)	37(5.9)		56(8.9)	2(0.3)
	OR (CI 95%)							1.83(1.35- 2.46)	9.07(2.71- 30.38)
Elimination Disorders	n (%)	50(3.3)	8(0.5)	332(22.7)	298(19.9)	93(6.1)	56(3.9)		2(0.1)
							
Eating Disorders	n (%)	4(25)	0	7(43.8)	3(20)	0	2(15.4)	2(12.5)	

## References

[1] Wittchen H-U (2004). Continued needs for epidemiological studies of mental disorders in the community. Psychotherapy and psychosomatics.

[2] MacMahon B, Pugh TF (1970). Epidemiology: principles and methods. Epidemiology: principles and methods.

[3] Smelser NJ, Baltes PB (2001). International encyclopedia of the social & behavioral sciences.

[4] Wittchen HU (2000). Epidemiological research in mental disorders: Lessons for the next decade of research—The NAPE Lecture 1999. Acta Psychiatr Scand.

[5] Bobevski I, Rosen A, Meadows G (2017). Mental health service use and need for care of Australians without diagnoses of mental disorders: findings from a large epidemiological survey. Epidemiol Psychiatr Sci.

[6] Shanfi V, Mojtabai R, Shahnvar Z, Alaghband-Rad J, Zarafshan H, Wissow L (2016). Child and Adolescent Mental Health Care in Iran: Current Status and Future Directions. Arch Iran Med.

[7] Polanczyk GV, Salum GA, Sugaya LS, Caye A, Rohde LA (2015). Annual Research Review: A meta‐analysis of the worldwide prevalence of mental disorders in children and adolescents. J Child Psychol Psychiatry.

[8] Fombonne E (1994). The Chartres study: I. Prevalence of psychiatric disorders among French school-aged children. Br J Psychiatry.

[9] Kroes M, Kalff AC, Kessels AG, Steyaert J, Feron FJ, Van Someren AJ (2001). Child psychiatric diagnoses in a population of Dutch schoolchildren aged 6 to 8 years. Journal of the American Academy of Child & Adolescent Psychiatry.

[10] Angold A, Erkanli A, Farmer EM, Fairbank JA, Burns BJ, Keeler G (2002). Psychiatric disorder, impairment, and service use in rural African American and white youth. Arch Gen Psychiatry.

[11] Canino G, Shrout PE, Rubio-Stipec M, Bird HR, Bravo M, Ramirez R (2004). The dsm-iv rates of child and adolescent disordersin puerto rico: prevalence, correlates, service use, and the effects of impairment. Arch Gen Psychiatry.

[12] Fleitlich-Bilyk B, Goodman R (2004). Prevalence of child and adolescent psychiatric disorders in southeast Brazil. J Am Acad Child Adolesc Psychiatry.

[13] Petersen DJ, Bilenberg N, Hoerder K, Gillberg C (2006). The population prevalence of child psychiatric disorders in Danish 8–to 9–year–old children. Eur Child Adolesc Psychiatry.

[14] Lynch F, Mills C, Daly I, Fitzpatrick C (2006). Challenging times: prevalence of psychiatric disorders and suicidal behaviours in Irish adolescents. J Adolesc.

[15] Leung PW, Hung S-f, Ho T-p, Lee C-c, Liu W-s, Tang C-p (2008). Prevalence of DSM-IV disorders in Chinese adolescents and the effects of an impairment criterion. Eur Child Adolesc Psychiatry.

[16] Pillai A, Patel V, Cardozo P, Goodman R, Weiss HA, Andrew G (2008). Non-traditional lifestyles and prevalence of mental disorders in adolescents in Goa, India. Br J Psychiatry.

[17] Heiervang E, Stormark KM, Lundervold AJ, Heimann M, Goodman R, Posserud M-B (2007). Psychiatric disorders in Norwegian 8-to 10-year-olds: an epidemiological survey of prevalence, risk factors, and service use. J Am Acad Child Adolesc Psychiatry.

[18] Frigerio A, Rucci P, Goodman R, Ammaniti M, Carlet O, Cavolina P (2009). Prevalence and correlates of mental disorders among adolescents in Italy: the PrISMA study. Eur Child Adolesc Psychiatry.

[19] Merikangas KR, Nakamura EF, Kessler RC (2009). Epidemiology of mental disorders in children and adolescents. Dialogues Clin Neurosci.

[20] Goodman R, Slobodskaya H, Knyazev G (2005). Russian child mental health a cross-sectional study of prevalence and risk factors. Eur Child Adolesc Psychiatry.

[21] Anselmi L, Fleitlich-Bilyk B, Menezes AMB, Araújo CL, Rohde LA (2010). Prevalence of psychiatric disorders in a Brazilian birth cohort of 11-year-olds. Soc Psychiatry Psychiatr Epidemiol.

[22] Benjet C, Borges G, Medina‐Mora ME, Zambrano J, Aguilar‐Gaxiola S (2009). Youth mental health in a populous city of the developing world: results from the Mexican Adolescent Mental Health Survey. J Child Psychol Psychiatry.

[23] Kessler RC, Avenevoli S, Costello EJ, Georgiades K, Green JG, Gruber MJ (2012). Prevalence, persistence, and sociodemographic correlates of DSM-IV disorders in the National Comorbidity Survey Replication Adolescent Supplement. Arch Gen Psychiatry.

[24] Ford T, Goodman R, Meltzer H (2003). The British child and adolescent mental health survey 1999: the prevalence of DSM-IV disorders. J Am Acad Child Adolesc Psychiatry.

[25] Vicente B, Saldivia S, de la Barra F, Kohn R, Pihan R, Valdivia M (2012). Prevalence of child and adolescent mental disorders in Chile: a community epidemiological study. J Child Psychol Psychiatry.

[26] Abolfotouh MA (1997). Behaviour disorders among urban schoolboys in south-western Saudi Arabia.

[27] Al-Kuwaiti MA, Hossain MM, Absood GH (1995). Behaviour disorders in primary school children in Al Ain, United Arab Emirates. Annals of tropical paediatrics.

[28] Swadi H (1998). Screening for psychiatric morbidity among a community sample of Arab children in the United Arab Emirates. Emirates Medical Journal.

[29] Mousa Thabet A, Vostanis P (2001). Epidemiology of child mental health problems in Gaza Strip.

[30] Eapen V, Jakka ME, Abou-Saleh MT (2003). Children with psychiatric disorders: The Al Ain community psychiatric survey. Can J Psychiatry.

[31] Alyahri A, Goodman R (2008). The prevalence of DSM-IV psychiatric disorders among 7–10 year old Yemeni schoolchildren. Soc Psychiatry Psychiatr Epidemiol.

[32] Mohammadi MR, Ahmadi N, Salmanian M, Asadian-Koohestani F, Ghanizadeh A, Alavi A (2016). Psychiatric disorders in Iranian children and adolescents. Iran J Psychiatry.

[33] https://www.amar.org.ir/Portals/0/census/1395/results/ch_nsonvm_95.pdf..

[34] Sepanlou SG, Parsaeian M, Krohn KJ, Afshin A, Farzadfar F, Roshandel G (2017). Disability-adjusted life-years (DALYs) for 315 diseases and injuries and healthy life expectancy (HALE) in iran and its neighboring countries, 1990â 2015: Findings from global burden of disease study 2015. Archives of Iranian medicine.

[35] Mohammadi MR, Ahmadi N, Kamali K, Khaleghi A, Ahmadi A (2017). Epidemiology of Psychiatric Disorders in Iranian Children and Adolescents (IRCAP) and Its Relationship with Social Capital, Life Style and Parents' Personality Disorders: Study Protocol. Iran J Psychiatry.

[36] Kaufman J, Birmaher B, Brent D, Rao U, Flynn C, Moreci P (1997). Schedule for affective disorders and schizophrenia for school-age children-present and lifetime version (K-SADS-PL): initial reliability and validity data. J Am Acad Child Adolesc Psychiatry.

[37] Ghanizadeh A, Mohammadi MR, Yazdanshenas A (2006). Psychometric properties of the Farsi translation of the kiddie schedule for affective disorders and schizophrenia-present and lifetime version. BMC psychiatry.

[38] Wittchen H-U, Nelson CB, Lachner G (1998). Prevalence of mental disorders and psychosocial impairments in adolescents and young adults. Psychol Med.

[39] Zarafshan H, Mohammadi M-R, Salmanian M (2015). Prevalence of anxiety disorders among children and adolescents in Iran: a systematic review. Iran J Psychiatry.

[40] Qin P, Agerbo E, Mortensen PB (2003). Suicide risk in relation to socioeconomic, demographic, psychiatric, and familial factors: a national register–based study of all suicides in Denmark, 1981–1997. Am J Psychiatry.

[41] Vega WA, Kolody B, Aguilar-Gaxiola S, Alderete E, Catalano R, Caraveo-Anduaga J (1998). Lifetime prevalence of DSM-III-R psychiatric disorders among urban and rural Mexican Americans in California. Arch Gen Psychiatry.

[42] La Maison C, Munhoz TN, Santos IS, Anselmi L, Barros FC, Matijasevich A (2018). Prevalence and risk factors of psychiatric disorders in early adolescence: 2004 Pelotas (Brazil) birth cohort. Soc Psychiatry Psychiatr Epidemiol.

[43] Kandemir G, Hesapcioglu ST, Kurt ANC (2018). What Are the Psychosocial Factors Associated With Migraine in the Child? Comorbid Psychiatric Disorders, Family Functioning, Parenting Style, or Mom’s Psychiatric Symptoms?. Journal of child neurology.

[44] Tonna M, Amerio A, Stubbs B, Odone A, Ghaemi SN (2015). Comorbid bipolar disorder and obsessive-compulsive disorder: A child and adolescent perspective. Aust N Z J Psychiatry.

[45] Deepmala, Coffey B (2014). Challenges in Psychopharmacological Management of a Young Child with Multiple Comorbid Disorders, History of Trauma, and Early-Onset Mood Disorder: The Role of Lithium. J Child Adolesc Psychopharmacol.

[46] Dilsaver SC, Akiskal HS, Akiskal KK, Benazzi F (2006). Dose–response relationship between number of comorbid anxiety disorders in adolescent bipolar/unipolar disorders, and psychosis, suicidality, substance abuse and familiality. J Affect Disord.

[47] Moss HB, Lynch KG (2001). Comorbid disruptive behavior disorder symptoms and their relationship to adolescent alcohol use disorders. Drug Alcohol Depend.

[48] Tuisku V, Pelkonen M, Kiviruusu O, Karlsson L, Marttunen M (2012). Alcohol use and psychiatric comorbid disorders predict deliberate self-harm behaviour and other suicidality among depressed adolescent outpatients in 1-year follow-up. Nord J Psychiatry.

[49] Moaddab M, Mangone E, Ray MH, McDannald MA (2017). Adolescent alcohol drinking renders adult drinking BLA-dependent: BLA hyper-activity as contributor to comorbid alcohol use disorder and anxiety disorders. Brain Sci.

[50] Brand-Gothelf A, Leor S, Apter A, Fennig S (2014). The impact of comorbid depressive and anxiety disorders on severity of anorexia nervosa in adolescent girls. J Nerv Ment Dis.

[51] Noterdaeme M, Schlamp D, Linder M, Kischel K-H (2004). Analyse der komorbiden psychiatrischen Diagnosen anhand der Basisdokumentation der Kinder-und Jugendpsychiatrie. Psychiat Prax.

